# Making Paving Stones from Copper Mine Tailings as Aggregates

**DOI:** 10.3390/ijerph17072448

**Published:** 2020-04-03

**Authors:** Elizabeth J. Lam, Vicente Zetola, Yendery Ramírez, Ítalo L. Montofré, Franco Pereira

**Affiliations:** 1 Chemical Engineering Department, Universidad Católica del Norte, Antofagasta CP 1270709, Chile; yendery.ramirez@ucn.cl (Y.R.); franco.pereira@alumnos.ucn.cl (F.P.); 2 Construction Management Department, Universidad Católica del Norte, Antofagasta C.P. 1270709, Chile; vzetola@ucn.cl; 3 School of Engineering Sciences, Lappeenranta-Lahti University of Technology, FI-53851 Lappeenranta, Finland; 4 Mining Business School, ENM, Universidad Católica del Norte, Antofagasta CP 1270709, Chile; imontofre@ucn.cl; 5 Mining and Metallurgical Engineering Department, Universidad Católica del Norte, Antofagasta CP 1270709, Chile

**Keywords:** tailing bricks, copper mine tailings, mine tailing stabilization, mining environmental liabilities

## Abstract

Copper mining, the central axis of Chile’s economic development, produces a large number of tailings, which become a potential environmental risk. This study aims to evaluate the mechanical properties resulting from the making of Portland cement mixtures with tailings as aggregates so that they can be eventually used in paving stones for building inactive tailings dams. Tailings coming from two dams at a concentration plant located in Taltal (Chile) were used. Currently, Dam 1 is inactive, while Dam 2 is active. The tailings samples obtained from both dams were granulometrically characterized by sieving. In addition, pH, humidity, Eh, and mineralogical assays (sulfides, oxides, sulfates, carbonates, phosphates, and silicates) were measured. The fines content of the tailings from Dams 1 and 2 with a sieve size of N°200 ASTM were 76.2% and 29.6%, respectively. Therefore, owing to their high percentage of fines, they cannot be as used as concrete aggregates. Aggregates must contain a maximum percentage of fines so that mortars and concrete can meet Chilean standards. In this paper, to comply with a 7% and 15% fines content lower than 0.075 mm, tailings materials were mixed with conventional aggregates containing very little fines. In addition, a reference mixture was made with only tailings aggregates with and without a superplasticizer additive. To measure the mixtures of cement, aggregates, and tailings, bending and compression strength assays were made of the specimens after a 28-day curing, according to the Chilean standard. The results of the study show that the addition of only part of the tailings to the mixture increases bending strength by 26% and compression strength by 180% compared with the reference mortar, with a fines content lower than 0.075 mm in the 7% mixture, thus allowing paving stone manufacture with tailings materials. In addition, it was possible to increase the workability of the reference mixture by using superplasticizers as additives.

## 1. Introduction

Chile is the world’s largest copper producer, a fact that has brought about a serious environmental impacts associated with risks to the environment and the population. Among these serious risks is the presence of mining environmental liabilities, mainly consisting of tailings dams, some of them active and others inactive. Some of the latter are abandoned. Tailings storage, for decades in some cases, has been associated with a big environmental footprint, considering the space occupied and the time the storage remains. The penetration of physical and chemical substances in the ground poses huge additional challenges to stabilize the ground and avoid mining acid drainage [1].

The mining industry produces a great amount of solid waste such as mining tailings and rock waste, damaging the environment and human health. In addition, the mining industry is responsible for significant environmental damage such as atmospheric contamination by particles, toxic atmospheric emissions, and the exhaustion and contamination of surface and underground water resources [2,3]. Currently, stricter regulations and public awareness are challenging the mining industry to reduce, reuse, and recycle waste via ecological and profitable strategies.

Historically, tailings have been stored in terrestrial systems called dams and reservoirs. To optimize the final disposal conditions, new technologies have been developed to reduce their impact on the environment. These emerging technologies include tailings thickening and filtration and marine disposal, the latter technology being highly controversial and restricted in many countries [4]. 

Some technologies studied for mining tailings stabilization are in situ paste disposal, coverage systems, cement addition, mixing, and agglomeration. In the case of rock acid drainage treatment, permeable reactive barriers are used. The physical solidification of waste materials improves their engineering properties and chemical stabilization changes the chemical form of heavy metals, modifying their mobility [5]. The leaching of a porous medium is influenced by waste and the chemical composition of the leaching medium, waste engineering and physical properties—that is, hydraulic conductivity, porosity and particle size, and the hydraulic gradient of the waste [6]. Mining tailings of sulfide deposits usually contain high concentrations of toxic elements whose mobility represents a potential environmental risk for surrounding ecosystems [7,8].

Mine tailings may contain high concentrations of sulfides and sulfates, which may generate sulfuric acid owing to the oxidation of sulfide minerals and leaching of associated metals from rocks containing sulfides when they are exposed to air and water. Due to the salinity usually present in tailings, metals such as Cu, Fe, and Mn show high mobility [2].

Particularly, mine tailings in Chile have been historically abandoned without any proper management due to inadequate legislation. Currently, the legislation requires that tailings be physically and chemically stabilized. Mining companies are forced to have a closure plan to ensure tailings landfill stability to protect the surrounding environment and the local population health [2]. 

Several studies have been conducted to make building bricks from waste materials: Ahmari and Zhang [9] studied the feasibility of using copper mine tailings to produce bricks using geopolymerization technology, without using clay and shale and requiring a lower temperature for kiln firing. Geopolymers are inorganic synthetic polymers made of aluminosilicates made up of minerals with Al_2_O_3_ and SiO_2_ content [10,11]. Among aluminosilicates are feldspar, chlorites, clay minerals, and some types of pozzolana. Geopolymers have become highly interesting as Portland cement substitutes owing to their low CO_2_ emission during production, high chemical and thermal resistance, and good mechanical properties at both ambient and extreme temperatures [1], with their use being interesting in the Atacama Region (Chile); Chen et al. [12] utilized hematite tailings with clay and Class F fly ash to produce bricks requiring up to 84% tailings weight; Chou et al. [13] used Class F fly ash to replace part of the clay and shale to produce bricks by the conventional method, with up to 40% fly ash in test runs on a commercial basis, with properties exceeding ASTM specifications; Morchhale et al. [14] produced bricks with copper mine tailings and ordinary Portland cement, by compressing the mixture in a mold. These bricks have a higher compressive strength and lower water absorption when the ordinary Portland cement content increases. Roy et al. [15] used gold mill tailings to produce bricks by mixing them with ordinary Portland cement, black cotton soils, or red soils. The ordinary Portland cement–tailings bricks were cured by submerging them into water, but the soil–tailings bricks were sundried and fired at high temperatures (<950 °C). Shon et al. [16] used stockpiled circulating fluidized bed combustion ash with Type I cement, lime, Class F fly ash, or calcium chloride to manufacture compressed bricks, with a 55.2 compaction pressure, placing the specimens at 23 °C and 100% relative room humidity for one day before air curing at room temperature.

Additionally, several techniques have been used for developing mine tailings bricks, each one with its advantages and disadvantages. Several methods using waste to make bricks require baking in a high-temperature oven or using alkaline solutions for geopolymers. In many applications, geopolymer offers a performance comparable to ordinary Portland cement, with several advantages such as the rapid development of mechanical strength, high acid resistance, expansion related to the reaction without and with the low dioxide content of alkaline silicon, excellent adhesion to aggregates, immobilization of toxic and hazardous materials, and low greenhouse gas emissions [9,17,18,19,20,21]. Curing temperature is an essential factor affecting geopolymerization and, thus, the strength of geopolymer brick specimens. For the copper mine tailings studied by [9], the optimum curing temperature is around 90 °C. Therefore, they still show the disadvantages of high energy consumption and high greenhouse gas emissions [9].

Ahmari and Zhang [9] studied the effects of sodium hydroxide solution concentration, water content, forming pressure, and curing temperature on the physical and mechanical properties of geopolymer bricks made of copper mine tailings. Conventional brick production requires clay, shale, and high kiln firing temperatures (900–1000 °C) [9]. Clay and shale are experiencing a shortage in several parts of the word. Additionally, the quarrying operation required to obtain clay and shale is energy-intensive, releasing a high volume of waste and impacting on the landscape [9].

Chen et al. [12] made building bricks using secondary resources such as 84% hematite tailings with clay and fly ash to improve the mechanic strength of the bricks and reduce bulk density, by mixing, forming, drying, and firing (980–1030 °C for 2 h). The main disadvantage of hematite tailings for making bricks is the presence of excess iron and little silica, with compressive strength decreasing with the addition of fly ash [12].

Numerous studies reveal the significant influence of the physical (specific gravity, particle shape, size, texture, size distribution, porosity) and chemical (mineralogy, active clay content) properties of a filler on primary pavement distresses (rutting, fatigue, low-temperature cracking, aging, and moisture susceptibility) [22,23,24,25,26]. Choudhary et al. [22] investigated waste materials as fillers in a graded bituminous macadam mix, i.e., copper tailings, carbide lime, brick dust, rice straw ash, red mud, limestone dust, and glass powder which, when mixed with fines, had a superior stiffness and cracking resistance, while mixtures with calcium predominance presented superior adhesion and moisture resistance. Fines filler particles influence the physical (porosity, size distribution, texture, size, particle shape, specific gravity) and chemical (active clay content, mineralogy) properties of primary pavement distresses [22]. The use of wastes as fillers can limit the consumption of conventional fillers, providing an effective solution for waste disposal [22].

A cemented tailings backfill or cemented paste backfill mixture is used as a supporting structure in underground mining operations, where the main components are mine tailings and binder [27]. Xu et al. [27] analyzed the tailings effect on the solid content, the type of cement reagent, and the effect of the binder proportion on unconfined compression strength and microstructure evolution.

A higher solid content increases the compactness of mine tailings; mechanical strength is proportional to the amount of binder content, with the pore and hydrated material distribution influencing solid content, binder type, and curing time (Xu et al., 2018) [27].

Nehdi and Tariq [6] studied Class C fly ash and cement kiln sulfate-resistant cement in the process of the stabilization–solidification of polymetallic sulfidic mine tailings, analyzing the leaching properties, hydraulic conductivity, and compression strength. Sulfidic tailings waste disposal is site-specific and involves a mine tailings facility, waste characterization, and site characterization and selection [6]. 

According to the National Geology and Mining Service (SERNAGEOMIN, for its acronym in Spanish), there are 740 tailings, 469 of which are inactive and 170 abandoned, the latter being the greatest threat due to their physical and chemical instability. Apart from this, mining producers in some Chilean regions create tailings via small mineral processing plants, which are deposited on unsafe sites owing to faulty legislation. Historically, most accidents and disasters in tailings are connected to or caused by physical stability failure, a fact that turns into a potential risk in Chile because it is a highly seismic country. One way of reducing instability in these inactive and abandoned tailings is utilizing them as inputs to make mixtures for building materials, thus encapsulating the heavy metals present in the tailings. 

**Figure 1:**
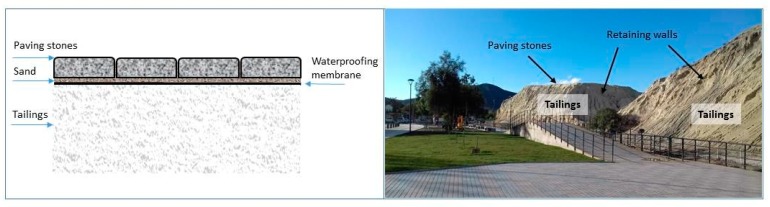
Scheme for using paving stones. The figure on the left shows a graphic. The figure on the right shows the proposed tailings surface coverage system at Andacollo commune.

To reduce the negative impact on the environment and use the secondary resource of copper mine tailings, this study provides an attractive method for recycling mine tailings into building materials and creating zero-emission tailings, which could be helpful for the production of construction bricks using green raw materials and industrial waste recycling [12,22]. Another way is to install a tailings surface coverage system, thus allowing us to decrease water infiltration and, therefore preventing the generation of acids and sulfide oxidation. This coverage system could be made with the mixtures obtained in this study, as shown in Figure 1.

The natural decrease in mineral content in the dams makes it necessary to process increasingly greater volumes and, therefore, produce bigger amounts of waste for the same production level. So, it is necessary to find methodologies to reduce risks associated with the presence of these wastes, some of which are being found in urban areas, as is the case in Andacollo in Chile [28]. This commune has nine big waste deposits in its urban section, which could destabilize at any moment since they are exposed to natural phenomena such as heavy rains and/or earthquakes, among others. The use of different waste materials as fillers instead of conventional material in densely graded bituminous macadam mixtures such as copper tailings could contribute to reducing the amount of Portland cement required [22]. 

The objective of this study is to develop a Portland cement-based mixture to be used in paving stones, with the physical and mechanical characteristics needed for its use, by mixing cement, as an agglomerate, with conventional aggregates (gravel and sand), tailings aggregates and water. Aggregates must consist of copper mine tailings in order to reuse them and reduce their impact on the environment. Additionally, this process to encapsulate tailings in concrete mixtures allows us to immobilize and insolubilize heavy metals present in tailings. Tailings used as inputs allow us to reduce the tailings volumes and the risks associated with them. From an economical viewpoint, tailings can be used as input for making building materials. The manufacture of concrete paving stones allows a reduction in contamination costs and levels. These paving stones may be used for the physical stabilization of the tailings, by using them on the upper part of the tailings. They could also be used in mixtures for making blocks to support their walls or for building tailings dams. 

This study contributes a methodology to develop new uses for mine tailings materials, which currently make up a potential environmental risk, by mixing tailings with conventional stone aggregates and examining the possibility of using superplasticizers as additives with tailings aggregates to improve their workability. 

## 2. Materials and Methods 

**Figure 2:**
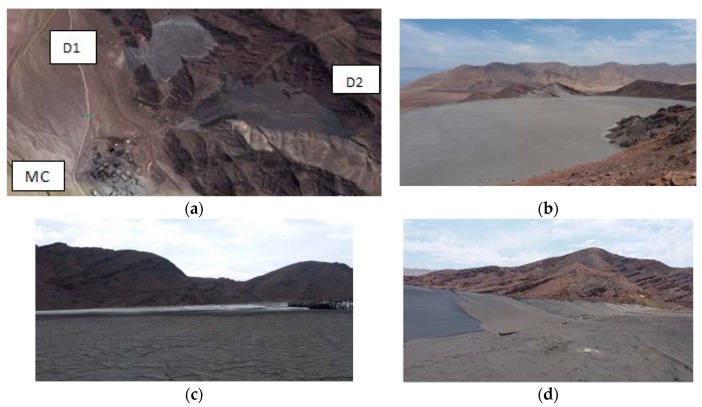
(**a**) Study area, tailings Dam 1 (D1) currently closed; (**b**) Dam 1 (close-up view); (**c**,**d**) tailings Dam 2 (D2).

This study focuses on the use of tailings aggregates for making Portland cement mixtures for paving stones from two tailings dams at a copper sulfide concentration plant located in Taltal commune, Antofagasta Region, Chile. Figure 2 shows the location of the mining deposit (MC) and the two tailings dams (D1 and D2) from which four surface samples (two from each dam) were extracted (beach zone), weighing 60 kg each, thus totaling 240 kg of tailings. Dam D1 is inactive, while Dam D2 is active and, therefore, still receives material.

Assays were conducted in the Materials Assay and Research Lab (LIEMUN, for its acronym in Spanish) at Universidad Católica del Norte (UCN) since it has all the equipment required for the study—that is, a mixer, a metal plate compaction table, and a machine for testing mortar compression and bending.

### 2.1. Analysis Method

#### 2.1.1. Mine Tailings Analysis

Tailings geocharacterization was conducted to obtain the main properties of the tailings and calculate the mixture design. The tailings material was used as an aggregate to make concrete mixtures.

a. Physical analysis

The percentage of tailings humidity was determined and the following assays were conducted, according to Chilean Norm (NCh), specifications: (1) determination by washing the material and passing it through mesh N° 200 (0.075 mm) in mineral aggregates—Standard NCh 1223 [29], (2) analysis by sieving the fines aggregates—standard NCh 165 [30], adapted to tailings conditions, and (3) actual density and absorption, NCh 1239 [31].

Particle size distribution was determined by sieving, according to Chilean standards [29,30], by making the following changes: the material retained in mesh N° 200, after washing, was dried at 110 ± 5 °C for 24 h. The sample was later passed through a set of sieves meeting ASTM international standards. Sieve sizes N° 16, 30, 50, 100, and 200 were used. The granulometric curve was obtained from these data to estimate the tailings–gravel–sand ratio to prepare the mixture.

b. Chemical analysis

To characterize tailings chemically, pH and Eh were measured potentiometrically in triplicate in a 1:9 tailings–distilled water ratio. 

c. Mineralogical Analysis

Quantitative evaluation of minerals by scanning electron microscopy (QEMSCAN) was used to analyze mineralogical mine tailings characterization. The results are expressed as a percentage of the total mass, as shown in Table 1. The tailings material was used for making concrete mixtures.

#### 2.1.2. Cement

CBB (Bío-Bío cement), a pozzolanic-type ordinary Portland cement was used. This type of cement is used due to its greater resistance to sulfates contained in the concrete.

#### 2.1.3. Conventional Aggregates 

Sand and gravel from “La Chimba” sector in Antofagasta were used. The sand is characterized as aeolian and the gravel as alluvial, with a 10 mm maximum size. Aggregates with a low content of fines smaller than mesh N° 200 (0.25%) were selected and mixed with tailings with a high fines content to provide a mixture with bigger particles. Sand and gravel granulometry was determined according to NCh 165 [30]. 

#### 2.1.4. Water

Tap water was used. It was not tested in the lab because it was considered suitable for concrete production, according to standard NCh 1498 [32].

#### 2.1.5. Superplasticizer Additive

A Sika Viscocrete 5100 polycarboxylate-based superplasticizer additive was used. The superplasticizer is an additive used as a highly effective reducer of the water necessary for making concrete mixtures manageable, thus increasing the initial and final concrete strength.

### 2.2. Cement Aggregate Mixture Assays

#### 2.2.1. Materials Dosage

A dosage used for ordinary mortars, NCh 158 [33], was used for the mixtures: two parts of cement (weight), six parts of aggregates, and one part of water. The water–cement ratio of the mixture was 0.5. In this study, the aggregates of the reference mixture were substituted by tailings. For this mortar, the water dosage was increased because the previous dosage made the sample dry and unworkable. To prepare a mixture with the ratios previously established and a 0.5 water–cement ratio, superplasticizers were added only to this mixture, with the dosage being 1.5% with respect to the cement weight. The same ratios were used for the dosages of gravel–sand–tailings mixtures, by using a certain quantity of tailings to obtain fines percentages of 7% and 15% from the aggregate mixture with mesh N° 200. Aggregate adjustment due to humidity was considered when preparing the mixture. 

#### 2.2.2. Reference Specimens

After characterizing the samples, three reference specimens were made for each dam, using tailings as aggregates. They were made according to Chilean standard NCh158 [32]—that is, 4 × 4 × 16 cm specimens [32]. The same procedure was followed for tailings from both dams. 

Table 2 shows the cement, water, and tailings dosages from Dams 1 and 2, used for making three reference specimens. The use of tailings with a big quantity of fines increased the amount of water required to maintain the sample’s workability, thus changing the water–cement ratio. To prove that the superplasticizer additive normally employed in concrete could be used in tailings mixtures, an assay was made with 1% additive. The dosage is shown in Table 3.

The method for specimen conservation required covering them with a non-absorbent plastic material to avoid water evaporation. Then, the mold was put into a wet chamber. The specimens were removed from the molds after 48 hours. The unmolded specimens were smoothly cleaned and classified. Once ready, the specimens were taken to the setting area and, later, to curing chambers for 28 days to ensure cement particle hydration. 

Each specimen was subjected to bending and compression strength assays, following Chilean standards [32]. Strength values are expressed in kg cm^−2^. The bending strength was determined for three specimens and compression strength for the corresponding six specimens. Both strengths, mortar and specimens, correspond to the mean value resulting from all assays.

#### 2.2.3. Gravel-Sand-Tailings Specimens

Specimens were made by using tailings as aggregates. The tailings were mixed with gravel and/or sand, so that the fines (mesh N° 200) in the aggregates incorporated could be 7% and 15%. Only tailings from Dam 2 were used because, according to size distribution, they contain a lower percentage of fine particles smaller than sieve size N° 200 (29.6%), thus making their use as inputs more attractive because the mortars obtained show better mechanical characteristics. The mortars were made in triplicate. Table 4 shows the compositions used in each case. 

To prepare 7% fines specimens, the following aggregate ratios were used: 25% tailings, 20% sand, and 55% gravel for a 1500 g total weight. To prepare 15% fines specimens, the following aggregate ratios were used: 52% tailings and 48% gravel for a total weight of 1500 g of aggregates. The ratios were obtained on the basis of tailings fines content below mesh N° 200 and the granulometry of the aggregates combined with tailings, gravel, and sand. 

## 3. Results and Discussions

### 3.1. Characterization

#### 3.1.1. Physical Characterization

**Figure 3:**
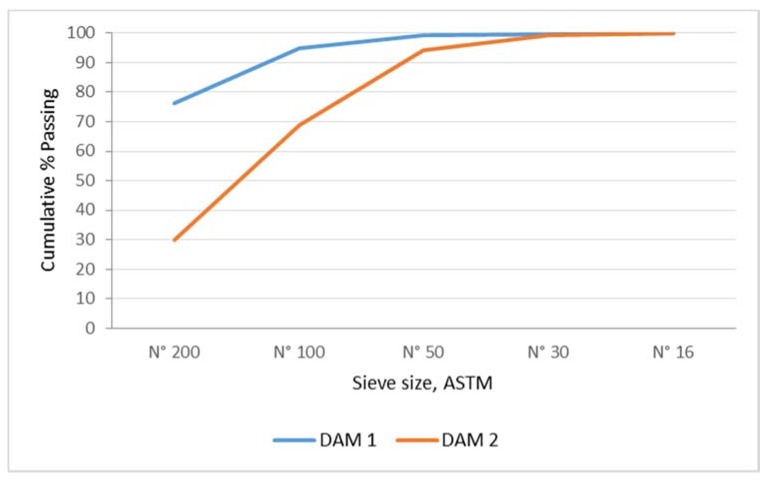
Particle size distribution of copper mine tailings samples.

Figure 3 shows the granulometric distribution of tailings. Dam 1 shows a higher ratio of fines particles compared with the samples from Dam 2—that is, 76.22% and 29.63%, respectively. For this reason, mine tailings from Dam 2 represent a better alternative as aggregates in the production of mortar, concrete, and paving stones for construction. As contents exceed standards, ways to reduce them must be found. This fines excess is responsible for significantly increasing the amount of water in the mixtures. The use of superplasticizer additives is one of the options, along with the use of other aggregates with less fines content.

For ground aggregates, Chilean standards indicate that the fines content must not be greater than 7% for fine aggregates and 1% for coarse aggregates. SERNAGEOMIN indicates that the percentage with sieve size N° 200 must be lower than 20% for the construction of deposit walls.

The real dry density of D1 and D2 tailings is 2420 and 2420 kg m^−3^, respectively. The water absorption of D1 and D2 is 2.70% and 2.57%, respectively.

**Figure 4:**
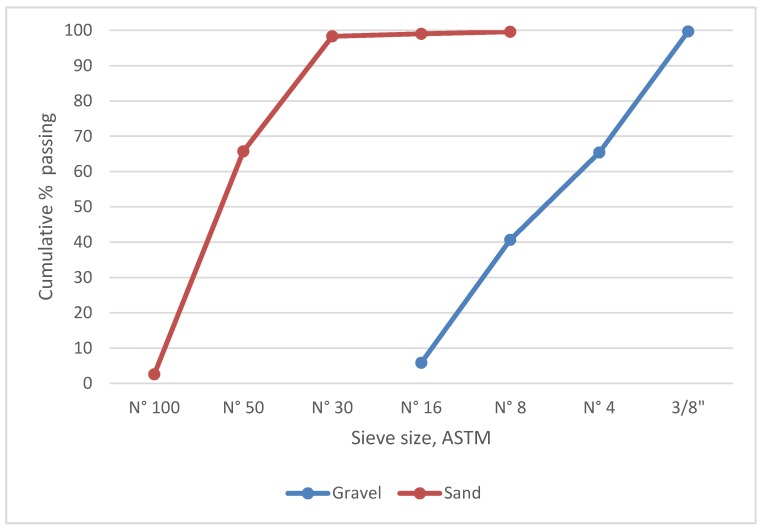
Granulometry of the sand and gravel used for gravel–sand–tailings mixtures.

Figure 4 shows the granulometry of the sand and gravel used for gravel–sand–tailings mixtures. The actual density (superficially saturated and dried) of the gravel and sand is 2730 and 2720 kg m^−3^, respectively. The gravel and sand water absorption is 1.43% and 0.53%, respectively. The amount of fines under mesh N° 200 for gravel and sand is 0.23 and 0.25 respectively.

#### 3.1.2. Chemical Characterization

The results of mine tailings sample measurements are shown in Table 5. The mine tailings pH is almost neutral. This reveals that it should not produce any effects on the mortar or concrete. Under conditions of low pH and high Eh, primary minerals actively oxidize and secondary soluble mineral phases dissolve [33]. D1’s average humidity is 9.90% and D2’s is 5.02% (kg of water and kg of tailings^−1^), considering five samples for each dam.

#### 3.1.3. Mineralogical Characterization

The mineralogical characterization of tailings was made with QEMSCAN. The most significant mineralogical contents for both dams were tectosilicates, with almost 50% mass, with the highest ratio being provided by Ca, Na feldspars, and quartz. The second most significant groups in abundance were phyllosilicates, the chlorite group contributing the most, with 21.32% and 15.6% for Dams 1 and 2, respectively. These values are quite important because quartz has a high mechanical resistance to impact and is mainly responsible for the mechanical strength of tailings products [12,34].

Sodium feldspar improves resistance to bending and impact; it increases resistance to stress; and provides hardness and durability, two characteristics that make the use of these tailings quite attractive as inputs for making paving stone mixtures. 

The amount of sulfates may exceed standards. So, the use of fewer amounts of this aggregate decreases the number of fines and sulfates in the mixture. 

#### 3.1.4. Mechanical Characterization

Table 6 shows the mean values of the results obtained from the bending strength assays made on the mortar by using tailings from D1 and D2. Since the sand was thoroughly replaced by tailings, the latter contain a high percentage of fines with sieve size N° 200—that is, 76.2% and 29.6% in tailings Dams 1 and 2, respectively. The aggregate and cement contents remained; however, the amount of water was increased until the proper consistency of the mixture was obtained. The mortar made from D1 tailings required 400 g of water—that is, 150 g greater than suggested by the ideal mixture, namely 60% greater than estimated. Meanwhile, the mortar made from D2 tailings as an aggregate, which contains a smaller amount of extremely fine particles, required 365 g of water—that is, 46% more than conventionally required. The mean strengths were obtained from two different sites in D1 and D2 tailings. The D1 tailings strength in Dams 1 and 2 was 44.37 and 42.46 kg cm^−2^, respectively, resulting in an average of 43.42 kg cm^−2^ for D1. For the mortar using raw material from D2, the average was 68.70 kg cm^−2^. These results reveal the influence of the percentage of fines on the bending strength of the mixtures since, in comparing mineralogy, it does not change as much between tailings.

The compression results of the mortars made with both tailings are shown in Table 7. Compression assays showed that D2 tailings are more resistant to bending stress than D1 tailings. The results of the compression assays indicate that the mixture using D2 tailings is more resistant to compression than the mixture made with D1 tailings, a fact that is also attributed to the greater presence of fines in D1 tailings.

The bending and compression results for tailings mortars with a superplasticizer are shown in Table 8 and Table 9. By reducing the water content, the strength increases. Nevertheless, the strengths obtained are small for making paving stones [31]. Table 9 shows the deviations for the value accepted in Chile, 500 kg cm^−2^ [35], determined as the ratio between the experimental and theoretical values, divided by the theoretical value multiplied by 100 (Δε). 

The mechanical assays conducted on specimens with cement–gravel–sand–tailings mixtures with a fines content smaller than sieve size N° 200, amounting to 7% and 15% in the aggregates, are shown in Table 8 for bending and Table 9 for compression. Results show that bricks made with 7% fines particles are more resistant to bending stress than those made with 15% fines particles, with sieve size N° 200. 

The results of the compression assays reveal that bricks made with aggregates containing 7% of fines particles smaller than 0.075 mm show a slightly greater compression strength than mixtures prepared with 15% of fines particles smaller than sieve size N° 200. The results obtained are slightly lower than indicated by [35], where individual strengths higher than 500 kg cm^−2^ are given. In the case of 7% fines the resulting average strength was a relative deviation of only −2.9%, while in the case of 15% fines, this deviation was higher, with an average value of −11.4%. The results in Table 9 correspond to mixtures with a higher docility than those used for making paving stones on an industrial basis. By using mixtures with either a smaller amount of water, a greater cement content or a superplasticizer, the most highly resistant paving stones could be made on an industrial basis.

## 4. Conclusions

In this study, copper mine tailings were used as aggregates for making cement–tailings and cement–gravel–sand–tailings mixtures to be used in the manufacture of paving stones for construction.

The cement–tailings mixtures did not reach the strengths required for making paving stones. On the other hand, tailings aggregates show fines contents higher than the standards with sieve size N° 200, thus greatly increasing water consumption. It is possible to add superplasticizer additives to the mixtures to reduce the amount of water required; however, although strengths increase when reducing the water/cement ratio, they are not enough to reach recommended values.

In using cement–gravel–sand–tailings mixtures, only material from D2 tailings could be utilized since D1 contained excess fines, thus making D1 ineffective.

The use of fines from 7% aggregates results in strengths 9% higher than using 15% aggregates; however, from an environmental and economic viewpoint, the second option is more attractive since it uses 53% of tailings—that is, more than twice the 7% alternative.

The potential use of tailings as part of building materials such as paving stones and blocks could greatly support physical and chemical tailings stabilization since they may be used as part of tailings walls and horizontal surfaces, thus reusing their waste, according to the circular economy.

The mine tailings from the two dams studied show a slightly neutral pH and contain a small quantity of sulfides. Thus, they do not represent a threat for developing acid mine drainage. The humidity contained in mine tailings is used to reduce water consumption during the production of paving stones from mine tailings.

## Figures and Tables

**Table 1:** Mineralogical tailings characterization.

Group	Mineral	Dam 1 Mass (%)	Dam 2 Mass (%)
Cu sulfides	Covellite	0.00	0.10
	Chalcocite/Digenite	0.002	0.09
Cu/Fe sulfides	Chalcopyrite	0.008	0.21
	Bornite	0.00	0.02
Cu oxidized minerals	Cuprite/Tenorite	0.01	0.03
	Other Cu Minerals	0.11	0.31
Gangue minerals containing Cu	Cu-bearing Phyllosilicates	2.80	2.09
	Cu-bearing Fe Oxide/Hydroxides	0.18	0.31
	Cu-bearing Wad	0.05	0.12
Sulfides	Pyrite	0.07	0.19
Oxides	Magnetite	3.09	8.23
	Hematite	4.50	8.32
	Goethite	0.56	0.65
	Other Fe Oxides/Hydroxides	1.00	0.98
	Ilmenite	0.58	0.46
	Rutile	0.24	0.29
	Corundum	0.00	0.01
Sulfates	Gypsum/Anhydrite/Bassanite	0.41	0.22
	Jarosite	0.01	0.02
	Alunite	0.01	0.02
Carbonates	Calcite/Dolomite	3.05	2.59
Phosphates	Apatite	1.01	0.90
Tectosilicates	Quartz	10.56	15.33
	K-Feldspars (Orthoclase, Anorthoclase)	4.83	4.88
	Ca, Na-Feldspars (Plagioclase Series)	33.67	26.25
Phyllosilicates	Kaolinite Group	0.30	0.46
	Biotite/Phlogopite	2.86	2.04
	Chlorite Group	11.53	7.08
	Muscovite/Sericite	3.26	4.25
	Illite	0.16	0.09
	Smectite Group (Montmorillonite, Nontronite)	3.15	1.60
	Pyrophyllite/Andalusite	0.06	0.10
Others ilicates	Hornblende	6.63	7.53
	Titanite	3.73	2.42
	Epidote	0.48	0.99
Others	Others	1.02	0.83
Total		100	100

**Table 2:** Cement, tailings, and water dosage for making reference specimens using materials from Dams 1 and 2.

Mortar Specimen	Dam 1 Mass (g)	Dam 2 Mass (g)
Cement	500	500
Water	400	365
Dry tailings	1500	1500

**Table 3:** Cement, tailings, water, and superplasticizer dosage for making reference specimens using materials from Dams 1 and 2.

Mortar Specimen	Dam 1 Mass (g)	Dam 2 Mass (g)
Cement	500	500
Water	250	264
Dry tailings	1500	1500
Superplasticizer	7.5	7.5

**Table 4:** Mortar mix with 7% and 15% fine particles from Dam 2 mine tailings.

Material	Mass (g) 7% Fines	Mass (g) 15% Fines
Water	250	250
Sand	300	0
Cement	500	500
Gravel	825	720
Dam 2	394	820

**Table 5:** Mine tailings sample measurement

Sample	pH	Eh Mvolt	Humidity (% kg_water_ kg_tailings_^−1^)
D1-Sample 1	7.28	−1200	10.69
D1-Sample 2	7.27	−1550	9.12
D2-Sample 1	6.99	−130	2.59
D2-Sample 2	7.05	−70	7.46

**Table 6:** Bending strength assay results after 28 days.

Bending	Strength (kN)	Pressure (kg cm^−2^)
D1-1	1.86	44.37
D1-2	1.78	42.46
D2-1	2.79	66.55
D2-2	2.97	70.84

**Table 7:** Compression strength assay results after 28 days.

Compression	Strength (kN)	Pressure (kg cm^−2^)
D1-1	14.81	94.33
D1-2	14.40	91.72
D2-1	27.00	172.24
D2-2	27.00	175.61

**Table 8:** Bending assay results for 7% and 15% fine particles.

Bending	7% Fine Particles		15% Fine Particles	
	Strength (kN)	Pressure (kg cm^−2^)	Strength (kN)	Pressure (kg cm^−2^)
D2-A	4.21	100.42	3.40	81.10
D2-B	3.75	89.45	3.42	81.34
D2-C	2.93	69.89	3.40	81.10

**Table 9:** Compression assay results for 7% and 15% fine particles.

Compression	7% Fine Particles			15% Fine Particles		
	Strength (kN)	Pressure (kg cm^−2^)	Δε %	Strength (kN)	Pressure (kg cm^−2^)	Δε %
D2-A1	73.57	468.72	−6.3	66.58	424.19	−15.2
D2-A2	78.97	503.12	0.6	66.96	426.61	−14.7
D2-B1	77.58	494.27	−1.1	70.19	447.18	−10.6
D2-B2	77.61	494.46	−1.1	70.37	448.33	−10.3
D2-C1	74.67	475.73	−4.9	72.25	460.31	−7.9
D2-C2	74.62	475.41	−4.9	70.64	450.05	−10.0
